# Food Addiction: Its Prevalence and Significant Association with Obesity in the General Population

**DOI:** 10.1371/journal.pone.0074832

**Published:** 2013-09-04

**Authors:** Pardis Pedram, Danny Wadden, Peyvand Amini, Wayne Gulliver, Edward Randell, Farrell Cahill, Sudesh Vasdev, Alan Goodridge, Jacqueline C. Carter, Guangju Zhai, Yunqi Ji, Guang Sun

**Affiliations:** 1 Discipline of Medicine, Faculty of Medicine, Memorial University of Newfoundland, St. John's, Canada; 2 Discipline of Laboratory Medicine, Memorial University of Newfoundland, St. John's, Canada; 3 Department of Psychology, Faculty of Science, Memorial University of Newfoundland, St. John's, Canada; 4 Discipline of Genetics, Faculty of Medicine, Memorial University of Newfoundland, St. John's, Canada; Pennington Biomedical Research Center, United States of America

## Abstract

**Background:**

‘Food addiction’ shares a similar neurobiological and behavioral framework with substance addiction. However whether, and to what degree, ‘food addiction’ contributes to obesity in the general population is unknown.

**Objectives:**

to assess 1) the prevalence of ‘food addiction’ in the Newfoundland population; 2) if clinical symptom counts of ‘food addiction’ were significantly correlated with the body composition measurements; 3) if food addicts were significantly more obese than controls, and 4) if macronutrient intakes are associated with ‘food addiction’.

**Design:**

A total of 652 adults (415 women, 237 men) recruited from the general population participated in this study. Obesity was evaluated by Body Mass Index (BMI) and Body Fat percentage measured by dual-energy X-ray absorptiometry. ‘Food addiction’ was assessed using the Yale Food Addiction Scale and macronutrient intake was determined from the Willet Food Frequency Questionnaire.

**Results:**

The prevalence of ‘food addiction’ was 5.4% (6.7% in females and 3.0% in males) and increased with obesity status. The clinical symptom counts of ‘food addiction’ were positively correlated with all body composition measurements across the entire sample (p<0.001). Obesity measurements were significantly higher in food addicts than controls; Food addicts were 11.7 (kg) heavier, 4.6 BMI units higher, and had 8.2% more body fat and 8.5% more trunk fat. Furthermore, food addicts consumed more calories from fat and protein compared with controls.

**Conclusion:**

Our results demonstrated that ‘food addiction’ contributes to severity of obesity and body composition measurements from normal weight to obese individuals in the general population with higher rate in women as compared to men.

## Introduction

Overweight and obesity are the abnormal or excessive accumulation of adipose tissue generally resulting from a chronic positive energy imbalance[1], [2]. Recently it has been shown that globally approximately 1.0 billion adults are overweight, and a further 475 million are obese [3]. In the United States, the prevalence of obesity among adults increased by 1.1% between 2007 and 2009. If this trend continues, by 2050 close to 100% of Americans will be overweight or obese [4]. Obesity and overweight are the fifth leading cause of global death [1] and the second most preventable cause of death in the United States [5]. Obesity is a complex multifactorial disease but the causes are not yet completely known[6]. Weight gain is usually the result of a complex interaction between an individual's biology and environmental factors which lead to energy surplus [7]. In westernized society, one of the main causes of a chronic energy surplus is a reduced physical activity level owing to a sedentary lifestyle. Another equally important cause of energy surplus is overeating [8], [9]. Overeating in some degree may occur in many individuals; however, a proportion may develop an obsessive/compulsive relationship to certain foods [10]. These individuals chronically consume more food than they need to maintain health and show compulsive intake behaviours associated with loss of control of eating [9], [11].

Accumulating research evidence has documented neurobiological and behavioural similarities between compulsive overeating and psychoactive drug dependence, leading researchers to use the term of ‘food addiction’ to describe this pattern of overeating [12]–[16]. In animal models, foods high in sugar and fat are particularly associated with addiction-like eating behaviour [17]–[19]. In human studies, it has also been suggested that the pattern of food intake in ‘food addiction’ may parallel substance dependence and this phenomenon might be understood with the same neurobiological, behavioral and clinical framework as conventional drug dependence [20]–[22]. Some researchers have argued that ‘food addiction’ should be included as a substance use disorder in the Diagnostic and Statistical Manual of Mental Disorders (DSM) [23], [24], although others have been critical of the clinical validity or utility of the ‘food addiction’ concept [9], [25]. Recently, the Yale Food Addiction Scale (YFAS) has been developed, and validated, as a tool for the diagnosis of ‘food addiction’ [26]–[28]. The YFAS criteria have been used to explore the prevalence of ‘food addiction’ in eating disorder patients [29], obese subjects [30] and junior college students [21]. There is a growing interest in the role of ‘food addiction’ in the increasing prevalence of human obesity which has reached an epidemic degree globally [14]. However, the exploration of ‘food addiction’ in humans is at an early stage and many fundamental questions are yet to be answered [25], [26].

First, the prevalence of ‘food addiction’ in the general population has not yet been assessed and this is an essential first step towards evaluating the potential contribution of ‘food addiction’ to human obesity. Only a few human studies are currently available and they were performed on specific cohorts like eating disorder patients [29], small stratified groups such as obese adults seeking weight loss [31] or junior college students [21]. However no data are currently available regarding the role of ‘food addiction’ in the general population and there seems to be a high proportion of ‘food addiction’ in obese with binge eating and obese seeking weight loss. However the association of ‘food addiction’ with BMI in junior college students was negligibly weak. Therefore, a second equally important question to be answered is whether ‘food addiction’ is significantly correlated with the severity of obesity in the general population. A third question concerns the intake of macronutrient in ‘food addiction’, because data suggest that each macronutrient may play a different role [32].

Hence the current study was designed to assess: 1) the prevalence of ‘food addiction’ in the Newfoundland population; 2) if the clinical symptom counts of ‘food addiction’ are significantly correlated with the severity of obesity in the general population; 3) if individuals classified as food addicted are significantly more obese than their non-food addicted counterparts; and 4) if food addicted subjects consumed more or less of any of the three macronutrients (i.e., fat, protein and carbohydrates).

## Materials and Methods

### Ethics Statement

This study was approved by the Health Research Ethics Authority (HREA), Memorial University of Newfoundland, Canada. All participants provided written informed consent.

### Study Sample

A total of 652 participants (415 female, 237 male) were recruited from the Canadian province of Newfoundland and Labrador (NL) via advertisements, posted flyers, and word of mouth. The inclusion criteria were: 1) age >19 years, 2) born in NL with family who lived in NL for at least three generations, 3) healthy without serious metabolic, cardiovascular or endocrine diseases, 4) not pregnant at the time of the study.

### Anthropometric Measurements

Body weight, height, waist and hip circumference were measured after a 12 hours fasting period. Subjects were weighed to the nearest 0.1 (kg) in a standard hospital gown on a platform manual scale balance (Health O Meter, Bridgeview, IL). A fixed stadiometer was used to measure height to the nearest 0.1 (cm). Hip circumference was measured with the flexible measuring tape to the nearest 0.1 (cm) at the level of largest circumference between the waist and thighs while the participant was in a standing position. The same procedure was used to measure waist circumference at the level of the umbilicus, midway between the lowest rib and iliac crest. BMI was calculated by dividing participants' weight in kilograms by the square of his/her height in meter (kg/m^2^). The subjects were classified as underweight/normal (BMI≤24.99) and overweight/obese (BMI≥25.00) based on BMI according to World Health Organization criteria [33].

### Body Composition Assessment

Whole body composition measurements including fat mass and lean body mass were measured using Dual-energy X-ray absorptiometry (DXA; Lunar Prodigy; GE Medical Systems, Madison, WI, USA). The measurements were performed in a supine position after 12 hours fasting. Total percent body fat (BF%) and percent trunk fat (TF%) were determined [34]. The subjects were also classified as under/normal weight and overweight/obese based on BF% according to the criteria recommended by Bray [35].

### ‘Food Addiction’ Assessment

The diagnosis of ‘food addiction’ was based on the Yale Food Addiction Scale (YFAS) [26]. This questionnaire consists of 27 items that assess eating patterns over the past 12 months. The YFAS translates the Diagnostic and Statistical Manual IV TR (DSM-IV TR) substance dependence criteria in relation to eating behavior (including symptoms such as tolerance and withdrawal symptoms, vulnerability in social activities, difficulties cutting down or controlling substance use, etc.) by applying the DSM-IV TR. The scale uses a combination of Likert scale and dichotomous scoring options. The criteria for ‘food addiction’ are met when three or more symptoms are present within the past 12 months and clinically significant impairment or distress is present. The Likert scoring option is used for food addiction symptom counts (e.g. tolerance and withdrawal) ranging from 0 to 7 symptoms [26], [29].

### Macronutrient intake and Physical Activity Assessment

Macronutrient intake (protein, fat and carbohydrate) during the past 12 months was assessed using the Willett Food Frequency Questionnaire (FFQ) [36]. Participants indicated their average use of a list of common food items, over the last 12 months. The amount of each selected food was converted to a mean daily intake value. The average daily intake for each food item consumed was entered into NutriBase Clinical Nutrition Manager (software version 9.0; CyberSoft Inc, Arizona). The total intake for each macronutrient per day was computed by the software for each subject [37]. The Baecke physical activity questionnaire was used to assess physical activity. This questionnaire assesses physical activity using three indices including work, sport and leisure [38].

### Statistical Analysis

Statistical analyses were performed using the R project for statistical computing version 2.15.2 (R Development Core Team). Data are presented as mean ± standard deviations (SD), maximum and minimum. Student t-test analyses were used to investigate the differences in measured variables between females and males. The prevalence of ‘food addiction’ was assessed in both the total cohort and different adiposity subgroups according to BMI and BF% by sexes. Relative risk ratios defined as the prevalence ratio were calculated to assess differences in the risk of ‘food addiction’ between sexes and between participants of different obesity status.

Student t-tests and Mann-Whitney-U tests (a non-parametric test) were employed to compare the anthropometric data related to obesity measures and macronutrients intake between ‘food addiction’ and non-food addiction groups. Furthermore, to take possible confounding factors into consideration, an ANCOVA was conducted to compare differences between food addicted and non-food addicted groups on obesity measurements with age, sex, smoking status, medication use and physical activity entered as covariates. Spearman partial correlation coefficients controlling for age, sex, smoking, medication use and physical activity were calculated to investigate the association between ‘food addiction’ and the severity of obesity. For all analyses, the alpha level was set at 0.05.

## Results

### Physical Parameters and Prevalence of ‘Food Addiction’

Demographic and physical characteristics of the participants are presented in Table 1. The prevalence of ‘food addiction’ according to the YFAS criteria was 5.4% in the entire population (in women and men it was 6.7% and 3.0%, respectively) (Table 2). When participants were classified as under/normal weight or overweight/obese based on BMI, the prevalence of ‘food addiction’ was 1.6% and 7.7% in these two groups respectively. When subjects were classified as under/normal weight or overweight/obese based on BF% the prevalence of ‘food addiction’ was 2.9% and 6.8%, respectively. The percentage of ‘food addiction’ significantly increased with increasing obesity status regardless of how adiposity was defined (RR = 0.21, p<0.001 and RR = 0.42, p = 0.03, respectively). When the samples were split based on gender, this trend remained significant only in females whose adiposity was classified using BMI (RR = 0.13, p<0.001). The prevalence of ‘food addiction’ was higher in women than in men (RR = 2.28, p = 0.046). Additionally, when using BMI adiposity classifications, but not the BF% adiposity classifications, overweight/obese women had higher prevalence of ‘food addiction’ as compared to overweight/obese men (RR = 3.50, p = 0.002).

**Table 1:** Characteristics of Study Participants*.

	Entire cohort	Female	Male
	Mean ± SD	Mean ± SD	Mean ± SD
	(Min–Max)	(Min–Max)	(Min–Max)
	n = 652	n = 415	n = 237
**Age (yr)** †	44.3±12.9	45.1±12.9	42.9±12.8
	(20–90)	(20–90)	(20–75)
**Height (cm)** †	168.4±9.1	163.3±5.9	177.3±6.6
	(147.7–196.6)	(147.7–187.9)	(156.8–196.6)
**Weight (kg)** †	78.1±18.3	71.5±15.7	89.8±16.6
	(46.6–149.8)	(46.6–149.8)	(57.1–149.5)
**BMI (kg/m^2^)** †	27.4±5.4	26.8±5.7	28.5±4.6
	(17.05–54.2)	(17.05–54.2)	(19.1–42.5)
**BF%** †	33.3±10.2	37.3±8.8	26.1±8.2
	(5.3–60.2)	(16.2–60.2)	(5.3–42.6)
**TF%** †	36.3±10.6	38.9±10.1	31.7±9.9
	(5.3–61.8)	(15.5–61.8)	(5.3–50.7)
**Waist (cm)** †	94.9±14.9	91.7±15.02	100.6±12.8
	(52–168)	(52–168)	(65–131)
**Hip (cm)**	100.5±12.1	100.2±13.2	100.9±9.9
	(74–155)	(74–155)	(79–134)
**Waist/Hip** †	0.9±0.08	0.9±0.07	1±0.05
	(0.68–1.62)	(0.68–1.62)	(0.75–1.14)

*Mean ± standard deviation (SD), (Maximum – Minimum), BMI–Body mass index, BF%–Percent body fat, TF%–Percent trunk fat.

† Significant difference between women and men (Independent t-test, p<0.05).

**Table 2:** Prevalence of ‘food addiction’ according to sex and obesity status*.

		Entire Cohort (%)	Female (%)	Male (%)	*Relative Risk*
**Entire Cohort**		5.4	6.7	3.0	2.28†
**Obesity status (BMI)**	Under/Normal Weight	1.6	1.5	1.9	0.81
	Overweight/Obese	7.7	11.4	3.3	3.50†
	*Relative Risk*	0.21‡	0.13‡	0.58	
**Obesity status (%BF)**	Under/Normal Weight	2.9	3.7	1.3	2.96
	Overweight/Obese	6.8	8.7	3.8	2.28
	*Relative Risk*	0.42‡	0.43	0.33	

*Prevalence of ‘food addiction’ (%), BMI–Body mass index and BF%–percent body fat. Obesity status (Under/Normal weight and Overweight/Obese) was defined by BMI and %BF according to the Bray [35] organization (WHO) criteria [33], respectively.

† Relative risk between females and males (Fisher's exact test, p<0.05).

‡ Relative risk between under/normal and overweight/obese groups (Fisher's exact test, p<0.05).

When food addicted subjects were classified by weight status based on BMI, 11.4% were under/normal weight, 88.6% were overweight/obese. When food addicted subjects were classified into adiposity group based on BF%, 20% were under/normal weight, 80% were overweight/obese (Table 3).

**Table 3:** The proportion of ‘food addiction’ according to obesity status*.

		Under/Normal weight% (n)	Overweight/Obese% (n)
**Food addition**	BMI	11.4% (4)	88.6% (31)
	BF%	20.0% (7)	80.0% (28)
**Entire Cohort**	BMI	38.2% (249)	61.8% (403)
	BF%	37.1% (242)	62.9% (410)

*Proportion of ‘food addiction’ (%), number of food addicts (n), BMI–Body mass index and BF%–percent body fat. Obesity status (Under/Normal weight and Overweight/Obese) was defined by BMI and %BF according to the Bray [35] and world health organization (WHO) [33] criteria, respectively.

### Correlations between clinical symptom counts of ‘food addiction’ and obesity

Spearman partial correlation coefficients controlling for sex and age were used to assess the relationship between the symptom counts of ‘food addiction’ and obesity measurements in the entire sample and in the non-food addicted subjects. All obesity related measurements (specifically markers related to central obesity) had strong positive correlations with YFAS symptom counts in both groups (Table 4). Furthermore, when we controlled for potential confounding factors including smoking, medication use and physical activity, the correlations remained significant.

**Table 4:** Correlation between ‘food addiction’ clinical symptom counts with obesity measurements*.

		BMI (kg/m^2^)	Weight (kg)	Hip (cm)	Waist (cm)	Waist/hip	Height (cm)	BF%	TF%
**Entire Cohort**	r	0.36	0.35	0.36	0.35	0.15	0.0091	0.31	0.32
	p	<0.001	<0.001	<0.001	<0.001	<0.001	0.82	<0.001	<0.001
**NFA**	r	0.32	0.30	0.31	0.30	0.12	0.007	0.27	0.28
	p	<0.001	<0.001	<0.001	<0.001	0.003	0.86	<0.001	<0.001

*NFA–non-food addiction, BMI–body mass index, BF%–percent body fat and TF%–percent trunk fat. Significance level for Spearman partial correlation (r) controlling for age and sex, were set to p<0.05.

### Comparison of obesity measurements and macronutrient intake between ‘food addiction’ and non- food addiction groups

Both student t-test and Mann- Whiney U test showed significant differences in all obesity measurements between ‘food addiction’ and non-food addiction groups (p<0.001) (Table 5). To take the other confounding factors into consideration, we conducted an ANCOVA controlling for sex, age, medication use, physical activity and smoking. All the differences remained significant. Food addicted subjects on average weighed 11.7 kg more and carried 4.6 more BMI than non- food addicted subjects. Additionally food addicted subjects had 8.2% greater body fat and 8.5% more trunk fat.

**Table 5:** Obesity measurements and macronutrient intake characteristics of ‘food addiction’ and non-food addiction*.

	FA	NFA	Mean difference	t test	Mann-Whitney-U test
	(Mean ± SD)	(Mean ± SD)		p value	p value
**BMI (kg/m^2^)**	31.8±6.6	27.2±5.2	4.6	<0.001	<0.001
**Weight (kg)**	89.2±21.5	77.5±17.9	11.7	0.003	<0.001
**Waist (cm)**	105.5±15.3	94.4±14.6	11.2	<0.001	<0.001
**Height (cm)**	167.2±9.4	168.5±9.1	−1.3	0.42	0.25
**Hip (cm)**	110.7±14.7	99.9±11.7	10.8	<0.001	<0.001
**BF%**	41.04±9.3	32.8±10.05	8.2	<0.001	<0.001
**TF%**	44.30±9.4	35.8±10.5	8.5	<0.001	<0.001
**Fat (g/kg)** †	0.80±0.4	0.8±0.7	0.005	0.95	0.59
**Carbohydrates (g/kg)** †	3.50±1.8	3.9±2.7	0.4	0.25	0.23
**Protein (g/kg)** †	1.20±0.5	1.2±0.9	−0.06	0.47	0.89
**Fat (%)** ‡	26.60±7.5	24.3±7.2	2.3	0.08	0.04
**Carbohydrate (%)** ‡	52.20±7.4	54.3±8.5	2.1	0.11	0.07
**Protein (%)** ‡	19.00±3.8	17.9±3.9	1.1	0.1	0.04

*Mean ± standard deviation (SD), FA–‘food addiction’, NFA–non-food addiction, BMI–body mass index, BF%–percent body fat and TF%–percent trunk fat. Independent t-test and Mann-Whitney-U test significance level was set to p<0.05.

† Macronutrient intake (g) per unit body weight (kg).

‡ Macronutrient intake (% total calorie intake).

Macronutrient intake was compared for the ‘food addiction’ and non-food addiction group (Table 5). Overall, the amount of macronutrients consumed, expressed as gram per kilogram of body weight, was not significantly different between the food addicted and non-food addicted participants. However, the percent calorie intake from protein (p = 0.04 from Mann-Whitney-U test and p = 0.03 from ANCOVA) and the percent calorie intake from fat (p = 0.04 from Mann-Whitney-U test, p = 0.11 from ANCOVA) was significantly higher in food addicted as compared to non-food addiction participants

## Discussion

In general, regardless of the various genetic predispositions and environmental influences, overeating is the primary factor responsible for the increasing prevalence of human obesity [14], [24]. To the best of our knowledge this is the first study reporting the contribution of ‘food addiction’ to the prevalence of human obesity in the general population [21], [29], [30]. One important finding is an estimation of the prevalence of ‘food addiction’ in the general Newfoundland population was at 5.4% (6.7% in women and 3.0% in men). In a previous study assessing obese patients with binge eating disorder (BED), the prevalence of ‘food addiction’ was reported to be as high as 56.8% [29], suggesting an overlap between binge eating and ‘food addiction’. The prevalence of ‘food addiction’ in obese individuals seeking weight loss treatment was 25%, while in another study obese subjects not seeking weight loss, the prevalence of ‘food addiction’ was 15.2% [30], [31]. In a cohort of junior college students with a normal BMI range, 8.8% met the YFAS criteria of ‘food addiction’; however the correlation between ‘food addiction’ clinical symptom counts and BMI was negligible [21], [39]. Our results indicated that 80–88.6% of food addicted individuals were overweight/obese based on Bray or BMI criteria providing strong evidence that ‘food addiction’ has contributed to the rising prevalence of obesity in the general population. Of note, food addicted individuals were also observed in the underweight and normal weight cohort, however in a lower number. The current findings suggest that obesity featured with ‘food addiction’ may represent an important subgroup of the obese with a distinctive aetiology. The identification of this subgroup will open a novel avenue to assess the aetiology of obesity and thus aid in finding new effective methods to treat and prevent obesity.

The subjects in the present study were recruited from the general Newfoundland population. The prevalence of overweight/obesity in the current study is similar to data reported from Health Canada on the province of Newfoundland (62.1%) [40]. The prevalence of ‘food addiction’ revealed in our study on the Newfoundland population may, to some degree, represent the prevalence in other Canadian provinces. Moreover our findings also suggests a potential difference between men and women in regards to ‘food addiction’, as overweight/obese women classified using BMI had a significantly higher rate of ‘food addiction’ as compared to men. This is similar to the case with eating disorders in which women also are significantly more likely to suffer from an eating disorder than men [41], [42]. Nevertheless larger studies in other populations are warranted to confirm the findings from our investigation.

The third major finding from the current study is the significant correlation between ‘food addiction’ and the severity of obesity in the general Newfoundland population. This finding appears to be robust as we were able to demonstrate this significant correlation throughout a number of analyses controlling for many confounding factors. Firstly, the clinical symptom counts of ‘food addiction’ was significantly correlated not only with BMI, but also with virtually all obesity related measurements including body weight, waist and hip circumferences, body fat and trunk fat percentage determined by DXA, an accurate measurement of body composition. This close correlation was seen in the non-food addicted group as well. We suggest that these robust and multiple correlations demonstrated a true association of ‘food addiction’ with human obesity. Additionally it was shown that obesity related variables were significantly different between food addicted and non-food addicted subjects. Participants who met criteria for ‘food addiction’ on average weighed 11.7 (kg) (25.79 lbs) more, had 4.6 higher BMI and possessed a 8.2% and 8.5% greater total body fat and trunk fat, respectively, as compared to non-food addicted subjects. These data provide the first direct evidence that ‘food addiction’ is strongly associated with obesity in the general population. Importantly, the individuals who met the criteria for ‘food addiction’ only represent between one fifth to one sixth of the total proportion of obese individuals in Newfoundland (25–30%) [40]. This suggests that ‘food addiction’ is likely an important factor in the development of human obesity but not the sole contributor.

Another important goal of our study was to examine differences in dietary patterns particularly macronutrients consumption between food addicted and non-food addicted subjects. Interestingly, the food addicted subjects' diet consisted of a higher percentage of calories from fat and protein, possibly suggesting that these types of foods are more likely to be associated with compulsive overeating. Given the significance of these findings will be important to verify these findings in other populations.

In the present study the YFAS was used as a diagnostic tool to classify participants with ‘food addiction’, as this set of measure and the criteria on which it is based have been validated [26]–[28]. Rather than directly asking if the subjects were addicted to food, the questionnaire assessed ‘food addiction’ based on DSM-IV-TR criteria [39]. Furthermore, using this set of criteria helped to distinguish subjects who regularly indulged in hyper palatable foods from those who have lost control over their eating behaviour [26].

One limitation of the present study was that the number of female participants was larger than the number of males. Given the sex difference in the prevalence of ‘food addiction’ found in the present study, it is possible that the actual prevalence in the general population may be lower than 5.4% if the study had consisted of equal numbers of women and men. Future studies using cohorts with an equal number of females and males in the population are warranted.

In summary, our study has revealed for the first time that: 1) the prevalence of ‘food addiction’ in the general Newfoundland population was 5.4%; 2) women are at high risk of ‘food addiction’ than men; 3) ‘food addiction’ contributes to human obesity and is significantly associated with the severity of obesity/amount of body fat from normal to obese individuals in the general population. Our findings provide strong evidence that ‘food addiction’ may represent a distinct aetiology of human obesity in the general population.
